# Nuclear Factor-Kappa B Family Member RelB Inhibits Human Immunodeficiency Virus-1 Tat-Induced Tumor Necrosis Factor-Alpha Production

**DOI:** 10.1371/journal.pone.0011875

**Published:** 2010-07-29

**Authors:** Michelle Kiebala, Oksana Polesskaya, Zhenqiang Yao, Seth W. Perry, Sanjay B. Maggirwar

**Affiliations:** 1 Department of Microbiology and Immunology, the University of Rochester School of Medicine and Dentistry, Rochester, New York, United States of America; 2 Department of Pathology and Laboratory Medicine, the University of Rochester School of Medicine and Dentistry, Rochester, New York, United States of America; 3 Center for Neural Development and Disease, Department of Neurology, the University of Rochester School of Medicine and Dentistry, Rochester, New York, United States of America; George Mason University, United States of America

## Abstract

Human Immunodeficiency Virus-1 (HIV-1)-associated neurocognitive disorder (HAND) is likely neuroinflammatory in origin, believed to be triggered by inflammatory and oxidative stress responses to cytokines and HIV protein gene products such as the HIV transactivator of transcription (Tat). Here we demonstrate increased messenger RNA for nuclear factor-kappa B (NF-κB) family member, transcription factor RelB, in the brain of doxycycline-induced Tat transgenic mice, and increased RelB synthesis in Tat-exposed microglial cells. Since genetic ablation of RelB in mice leads to multi-organ inflammation, we hypothesized that Tat-induced, newly synthesized RelB inhibits cytokine production by microglial cells, possibly through the formation of transcriptionally inactive RelB/RelA complexes. Indeed, tumor necrosis factor-alpha (TNFα) production in monocytes isolated from RelB deficient mice was significantly higher than in monocytes isolated from RelB expressing controls. Moreover, RelB overexpression in microglial cells inhibited Tat-induced TNFα synthesis in a manner that involved transcriptional repression of the TNFα promoter, and increased phosphorylation of RelA at serine 276, a prerequisite for increased RelB/RelA protein interactions. The Rel-homology-domain within RelB was necessary for this interaction. Overexpression of RelA itself, in turn, significantly increased TNFα promoter activity, an effect that was completely blocked by RelB overexpression. We conclude that RelB regulates TNFα cytokine synthesis by competitive interference binding with RelA, which leads to downregulation of TNFα production. Moreover, because Tat activates both RelB and TNFα in microglia, and because Tat induces inflammatory TNFα synthesis via NF-κB, we posit that RelB serves as a cryoprotective, anti-inflammatory, counter-regulatory mechanism for pathogenic NF-κB activation. These findings identify a novel regulatory pathway for controlling HIV-induced microglial activation and cytokine production that may have important therapeutic implications for the management of HAND.

## Introduction

Human Immunodeficiency Virus-1 (HIV-1) enters the central nervous system (CNS) early after infection, and in many cases may ultimately result in HIV-1 associated neurologic disease (HAND) [1]. HAND can include neurocognitive impairments, motor deficits, or dementias [2], and continues to be a significant source of morbidity despite efficacious reduction of viral load by comprehensive anti-retroviral therapy (cART) [3]–[10]. Traditionally, onset of HAND correlated with CNS viral load, and was principally subcortical in origin, with neuropathology including multinucleated giant cells, reactive astrocytosis, myelin pallor, reduced dendritic complexity and synaptic density and neuronal loss [11]–[16]. Recent neuropathologic reports of severe white matter damage (i.e. leukoencephalopathy) in patients with HIV-1 infection and on cART [17]–[23], including significant frontostriatal and prefrontal cortex involvement in HAND [24]–[27], suggest that additional patterns of primary brain disease are emerging, either due to alterations in host cell signaling, or as yet unexplained interactions between virus, vulnerable populations of neural cells, and cART [6], [28].

The pathogenesis of HAND likely arises from a toxic milieu of secretory neurotoxins released from HIV-1 infected, brain-resident mononuclear phagocytes and glia, and oxidative stress, which together adversely affect neuronal function. HIV productively infects microglia and perivascular macrophages, the resident phagocytes of the CNS, but does not infect neurons. This suggests that the neuropathology caused by HIV is indirect. Accordingly, neurologic deficits in HAND are more closely correlated with the presence of activated macrophages and microglia than with the amount of neuronal apoptosis or viral RNA [29]–[32]. Soluble viral proteins such as Tat and the glycoprotein gp120 can be released from infected microglia and macrophages [33]. Tat protein has been detected in blood plasma, serum, and cerebral spinal fluid (CSF) from HIV+ individuals, at levels ranging from 1–40 ng/ml [34], [35], thus local extracellular concentrations in the CNS may be much higher, particularly proximal to HIV+ pervivascular cells [36]. Tat also interacts with and activates neighboring, uninfected cells including microglia, astrocytes and neurons. Both infected and activated microglia and astrocytes produce the pro-inflammatory cytokines tumor necrosis factor-alpha (TNFα) and interleukin-1 beta (IL-1β), which further activate neighboring cells. Infected and activated cells also produce chemokines such as monocyte chemotactic protein-1 (MCP-1), attracting more inflammatory monocytes and macrophages [37]–[39]. Thus, circulating Tat has a high propensity to trigger this vicious cycle, leading to neurologic deficits [34].

HIV-1 Tat is necessary for viral replication and activates the nuclear factor-kappa B (NF-κB) signal transduction pathway in cells of the CNS [40], [41]. Cytokine and chemokine gene expression in microglia, in turn, is also NF-κB dependent [41]. The NF-κB family of transcription factors is intricately involved in both innate and adaptive immune responses as well as in inflammation. In mammalian cells, there are five NF-κB family members including RelA (p65), RelB, cRel, p52 (with its precursor p100), and p50 (with its precursor p105). Homo- and hetero-dimeric combinations of the five NF-κB proteins activate transcription of NF-κB target genes. In resting cells, NF-κB dimers are retained in the cytoplasm through their inhibitory interaction with the inhibitor of kappa B (IκB) molecules [42]. Signals leading to activation of the NF-κB pathway include the pro-inflammatory cytokines TNFα and IL-1β [43]. Initiation of the NF-κB pathway is characterized by signal-induced activation of the inhibitory κB kinase (IKK) complex. IKK then phosphorylates IκBα leading to its subsequent ubiquitination and proteolytic degradation [44]. Degradation of IκBα frees NF-κB dimers to move to the nucleus, where they can then activate transcription. We, and others, have demonstrated that Tat-induced production of cytokines (e.g. TNFα) by microglia involves activation of the NF-κB pathway, as evidenced by the rapid degradation of IκBα in Tat treated microglial cells [41], [45]. Indeed, overexpression of IκBα S32/36A, which is resistant to proteasomal degradation, blocks Tat-induced TNFα synthesis in microglial cells, thereby suggesting that Tat-induced TNFα synthesis is NF-κB dependent [45].

Here we show that Tat treatment of microglial cells induces *de novo* synthesis of RelB. The NF-κB family member RelB has many distinguishing features compared to the other NF-κB proteins, such as its unique amino-terminal leucine zipper motif [46]. RelB also preferentially interacts with p100 rather than the usual NF-κB inhibitory molecules (e.g. IκBα) [47], [48]. RelB has lower relative DNA binding activity at prototypical response elements, and can both activate and inhibit transcription of target genes [49], [50]. Specific nuclear DNA κB binding sites are efficiently recognized by RelB/p52 DNA heterodimers, allowing for transcription of certain κB-regulated target genes [49], [51]. RelB also forms inactive complexes with RelA [52]–[55]. In addition, studies in RelB^−/−^ mice have demonstrated impaired cellular immunity and development of multi-organ inflammation that involves activation of myeloid cells [56], [57]. RelB has also been shown to be inhibitory to the TNFα promoter in fibroblasts [58], [59]. Together these results strongly suggest that RelB has anti-inflammatory and cytokine-regulating properties. Herein we investigate these anti-inflammatory properties of RelB in the context of HIV-induced neuro-inflammation.

Initial experiments in transgenic mice expressing Tat under a doxycycline (Dox)-inducible glial fibrillary acidic protein (GFAP) promoter demonstrated increased RelB message in the brains of Dox-induced mice, confirming that Tat increases RelB *in vivo*. Furthermore, Tat also enhanced TNFα release from monocytes isolated from RelB^−/−^ mice as compared to monocytes from wild-type (WT) mice, which is in agreement with previous reports of anti-inflammatory properties of RelB. Next, in vitro experiments were conducted to determine the mechanisms of RelB inhibition of TNFα release from microglial cells. These experiments suggested that RelB binds RelA to inhibit RelA-dependent activation of the TNFα promoter, and that this RelB/RelA mechanism of interaction requires the entire RelB Rel Homology Domain (RHD). Taken together, these results suggest that RelB may regulate cytokine synthesis by counter-regulating NF-κB activated pathways, a finding that has important therapeutic implications for the management of HAND.

## Methods

### Reagents

Cycloheximide (CHX) and MG-132 were obtained from BioMol International/Enzo Life Sciences (Plymouth Meeting, PA, USA). CHX was used at concentrations of 10 and 25 µg/mL and MG-132 was used at a concentration of 50µM. Viral protein gp120 *SF162* was obtained from Immunodiagnostics (Woburn, MA, USA), and used at a concentration of 100nM. All other chemicals and regents were purchased from Sigma-Aldrich (St. Louis, MO, USA).

HIV-1 Tat 1–72 was expressed in Sf9 insect cells using a baculovirus system and was purified using Ni-NTA agarose according to the BD BaculoGold 6×His expression and purification protocol (BD Biosciences, San Jose, CA, USA). Briefly, the Tat 1–72 sequence (from HIV-1, YU-2 isolate) was PCR amplified and cloned into XhoI and NcoI sites in the pAcHLT-B baculovirus transfer vector. The recombinant plasmid was confirmed by sequencing. This transfer vector was then co-transfected into Sf9 cells along with BD BaculoGold linearized baculovirus backbone DNA. Resultant virus stock was propagated in Sf9 cells. Tat protein was then purified from lysates of infected Sf9 cells using Ni-NTA agarose beads.

Importantly, the Tat produced in this fashion and used for these studies was functionally indistinguishable from E. coli prepared Tat 1–72, and caused similar microglial activation of NF-κB related pathways as previously seen for E. coli Tat 1–72 (Text S1, Figure S1). Therefore this Sf9 Tat, hereafter referred to as “Tat,” was used for these studies, allowing us to take advantage of this more efficient protocol for producing HIV-1 Tat in the high yields required for these studies.

Tat 1–101 (Immunodiagnostics,Woburn, MA, USA), Tat 1–72 (Sf9-Tat), Tat 1–72 (E. coli Tat; kind gift of Dr. A. Nath, Johns Hopkins University, Baltimore, MD, USA), Tat C31S (Diatheva, Fano, Italy), Tat Δ31–61 (obtained from Philip Ray, University of Kentucky, Lexington, KY, USA) and Tat peptides (Tat 48–72: G-R-K-K-R-R-Q-R-R-R-P-P-Q-D-S-Q-T-H-Q-S-S-L-S-K-Q; GenScript, Piscataway, NJ, USA; Tat 46–60: S-Y-G-R-K-K-R-R-Q-R-R-R-P-P-Q; and Tat 65–80: H-Q-V-S-L-S-K-Q-P-T-S-Q-P-R-G-D; Intracel Corp., Cambridge, MA, USA) were used at 100nM (∼800 ng/ml) concentration unless otherwise described. It should be noted that soluble Tat levels in HIV patient sera have been measured up to 40 ng/mL [34], [35]. It has also been shown that Tat can interact with endogenous glycosaminoglycans and heparin sulfates, thereby potentially lowering its measurable concentration *in vivo* [60]. Therefore, it is likely that Tat concentrations surrounding HIV-infected cells are much higher [36]. In addition, Tat's exceedingly strong affinity for other proteins and glass/plastic, and its temperature susceptibility, make it impossible to determine exactly what fraction of the Tat “starting dose” actually reaches the experimental specimen, thus likely leading to underestimation of Tat functions in vitro [61]. Finally, Tat's effects in vivo are likely to occur over long-term exposures. Chronic, low dose, in vivo effects of any reagent are often appropriately modeled in vitro, by proportionately higher doses of that same reagent, over more acute time frames. For these reasons, and in order to be consistent with previous experiments from our laboratories, and others, we use 100nM Tat for these experiments, which is at the lower end of the Tat dose range seen with many comparable studies, and which many studies have found to be an appropriate Tat dose by which to model Tat's in vivo effects in vitro.

### Cell Cultures

#### Primary cultures

Human peripheral monocytes were isolated from commercially obtained soft-spun buffy coats (New York Blood Center, Long Island City, NY, USA) derived from HIV-1 and hepatitis B virus-seronegative donors, using immunomagnetic isolation methods as described [62], [63]. Briefly, peripheral blood mononuclear cells (PBMCs) were isolated from buffy coats after centrifugation on a lymphoprep gradient (AXIS-Shield, Oslo, Norway). Monocytes were then isolated by positive selection with anti-CD14 MACS beads (Miltenyi Biotec, Bergisch Gladbach, Germany; Auburn, CA, USA), and cultured in RPMI 1640 medium supplemented with 10% fetal bovine serum (FBS), and antibiotics for 18–24h before use. The monocyte cultures used in our experiments were >95% pure as determined by morphologic criteria.

Mouse monocytes were isolated from bone marrow derived from age-matched C57BL/6 (RelB^+/+^) or RelB^−/−^ mice by positive selection with mouse/human anti-CD11b MACS beads (Miltenyi Biotec, Bergisch Gladbach, Germany; Auburn, CA, USA). These cells were cultured in RPMI 1640 medium supplemented with 10% FBS and antibiotics for 16h before use.

Human astrocytes were kindly supplied by Dr. Anuja Ghorpade (University of North Texas Health Science Center, Fort Worth, Texas, USA). Briefly, brain tissue was mechanically dissociated, centrifuged, and resuspended in Dulbecco's modified Eagle medium (DMEM) supplemented with 10% FBS and 1× penicillin-streptomycin-glutamine for 7 days. Non-adherent microglia and oligodendrocytes were removed by extensive agitation (260 rpm) on a rotating platform. Cultures of astrocytes were >98% pure as determined by immunocytochemical (ICC) staining for GFAP (rabbit polyclonal antibody, Chemicon/Millipore, Billerica, MA, USA).

***Cell-lines:*** The murine microglial cell-line (BV-2) and human embryonic kidney cell line (HEK 293) were obtained from Dr. R. Donato (University of Perugia, Perugia, Italy) and American Type Culture Collection (ATCC, Manassas, VA, USA) respectively. These cells were maintained in DMEM containing 10% FBS, 2mM glutamine, and antibiotics, by standard procedures.

### ELISA

TNFα levels were measured in culture supernatants (pre-cleared by brief centrifugation) by using a mouse TNFα ELISA kit (eBioscience, San Diego, CA, USA) according to the manufacturer's instructions. This kit has a minimum sensitivity threshold of 8 pg/ml. Briefly, 50 µl of cell culture supernatant was incubated in a 96-well plate pre-coated with a TNFα-specific monoclonal antibody for 1.5h. After extensive washing, binding of TNFα was detected by incubation with biotinylated antibodies, followed by streptavidin-peroxidase; colorimetric enzyme assays were performed to detect bound TNFα. Other cytokine levels were measured using Bio-Plex Multi-Plex analysis for detecting a panel of multiple cytokines from a single sample (Bio-Rad, Hercules, CA, USA). Briefly, this technology uses multiple spectrally identifiable polystyrene beads, each coated with a different anti-cytokine antibody, followed by target, then secondary antibody binding, to detect multiple cytokines from a single sample in typical sandwich-assay fashion. This assay was utilized to measure levels of IL-1β, IL-6, and MCP-1 in supernatant samples, as previously described [64]–[66]. TNFα ELISA results were also confirmed by this assay.

### Electrophoretic Mobility Shift Assays

Nuclear extracts were prepared from BV-2 cells as previously described [67]. Electrophoretic mobility shift assays (EMSAs) were performed by incubating nuclear extracts with ^32^P-radiolabeled high affinity, double-stranded DNA probe suspended in EMSA reaction buffer (12mM HEPES, pH 7.9, 100mM NaCl, 0.25mM EDTA, 1mM dithiothreitol, and 1mM phenylmethanesulfonyl fluoride) at room temperature for 10 min, followed by resolution of the protein-DNA complexes on native 4% polyacrylamide gels, then autoradiography. The double stranded oligonucleotide probes used in EMSA were as follows: (1) NF-κB: 5′-CAACGGCAGGGGAATTCCCCTCTCCTT-3′ and (2) OCT-1: 5′-TGTCGAATGCAAATCACTAGAA-3′. For antibody supershift assays, 1 µg of antiserum recognizing each of the NF-κB subunits (Santa Cruz Biotechnologies Inc., Santa Cruz, CA, USA) was added to the EMSA reaction 10 min prior to electrophoresis. Specificity and reactivity of the antibodies was confirmed as described previously [68], [69].

### Plasmids

The luciferase reporter construct driven by NF-κB as well as the RelA and cRel plasmids were obtained from Dr. S. C. Sun (MD Anderson Cancer Center, University of Texas, Houston, TX, USA). The plasmids expressing mouse RelB (mRelB) and influenza hemagglutinin (HA)-tagged derivative of RelA (HA-RelA) were obtained from Dr. Bernd Baumann (Ulm University, Ulm, Germany). We PCR amplified the mRelB sequence from this plasmid and inserted it into the pcDNA3.1-Myc/His(-)B vector (Invitrogen, Carlsbad, CA, USA). The luciferase reporter construct driven by the mouse TNFα (mTNFα) promoter was obtained from Dr. Dmitry Kuprash (Engelhardt Institute of Molecular Biology, Moscow, Russia).

### Transient Transfections

HEK 293 cells were transfected with plasmid DNA using Lipofectamine 2000 (Invitrogen, Carlsbad, CA, USA). Cells were seeded at 1.8×10^5^ cells/well in 24-well plates 18h prior to transfection. Each transfection reaction contained a total of 0.85 µg plasmid DNA. After addition of the transfection mixture to the cells, they were incubated at 37°C for 24h. BV-2 cells were transfected with plasmid DNA using Lipofectamine LTX (Invitrogen, Carlsbad, CA, USA) or Nucleofector (Amaxa/Lonza, Basel, Switzerland; Walkersville, MD, USA). For Lipofectamine transfections, cells were seeded at 1×10^5^ cells/well in 24-well plates 18h prior to transfection, then transfected as for Lipofectamine 2000 above. For nucleofection, 5×10^6^ cells were transfected with 10 µg NF-κB luciferase plasmid DNA. Transfected cells were plated at 5×10^5^ cells/well in a 24-well plate and incubated for 24h prior to Tat treatment. Media was changed 4h after transfection and again prior to treatment to reduce cell toxicity. To determine nucleofection-based transfection efficiency, an aliquot of BV-2 cells was transfected with a GFP-expressing vector, pMax-GFP (Amaxa/Lonza, Basel, Switzerland; Walkersville, MD, USA), and 24h after transfection, greater than 90% of BV-2 cells were GFP-positive.

### Luciferase assays

A luciferase reporter plasmid containing NF-κB responsive elements upstream of a firefly luciferase gene was transfected into BV-2 cells using Nucleofector (Amaxa/Lonza, Basel, Switzerland; Walkersville, MD, USA). 24h post-transfection, cells were either left untreated or incubated for 8h with 100nM Tat. Cell lysates were prepared using reporter lysis buffer (Promega Life Sciences, Madison, WI, USA), and luciferase activity was measured. A luciferase reporter plasmid with the mouse TNFα promoter region upstream of a luciferase reporter was transfected into either HEK 293 cells using Lipofectamine (Invitrogen, Carlsbad, CA, USA) or BV-2 cells using Nucleofector (Amaxa/Lonza, Basel, Switzerland; Walkersville, MD, USA). 24h post-transfection, cell lysates were prepared using reporter lysis buffer (Promega Life Sciences, Madison, WI, USA), and luciferase activity was measured with a Lumicount Microplate Luminometer (Packard Instrument Company, Meriden, CT; now PerkinElmer, Waltham, MA). In these assays, total protein amount as determined by Bradford assay (Bio-Rad, Hercules, CA, USA), was used to normalize the samples.

### Immunoblotting assays

Following the indicated treatments, whole cell lysates were prepared in ELB buffer (50mM HEPES (pH 7), 250mM NaCl, 0.1% Nonidet P-40, 5mM EDTA, 10mM NaF, 0.1mM Na_3_VO_4_, 50µM ZnCl_2_, supplemented with 0.1mM PMSF, 1mM DTT, and a mixture of protease and phosphatase inhibitors). Cellular debris was removed by high-speed centrifugation. Lysates were fractionated on 7.5% SDS-PAGE gels and protein was electrophoretically transferred to Hybond ECL nitrocellulose membrane (GE Healthcare Bio-Sciences Corporation, Piscataway, NJ, USA). The membranes were analyzed for immunoreactivity with primary antibodies raised against RelB (1∶1000), RelA (1∶1000), IκBα (1∶1000), or α-Tubulin (1∶1000; all from Santa Cruz Biotechnologies Inc., Santa Cruz, CA, USA), RelA P-S276 (1∶1000), p100/p52 (1∶1000, Cell Signaling Technology, Danvers, MA, USA), Actin (1∶1000, Calbiochem/EMD Chemicals, Gibbstown, NJ, USA), or antiserum to HIV-1 Tat (1∶3000; NIH AIDS Research & Reference Reagent Program, Germantown, MD, USA). Bound antibodies were detected by species-specific, horseradish peroxidase (HRP)-conjugated secondary antibodies (1∶3000, GE Healthcare Bio-Sciences Corporation, Piscataway, NJ, USA), followed by addition of ECL reagent (Pierce Biotechnology/Thermo Fisher Scientific, Rockford, IL, USA) and subsequent exposure to x-ray film. Equal loading and uniformity of protein transfer to the nitrocellulose membrane were verified by stripping and reprobing the membranes with primary antibodies specific to α-Tubulin or Actin.

### Tat transgenic mice and Real-Time RT-PCR analyses

Tat transgenic mice were kindly provided by Dr. Johnny He (Indiana University School of Medicine, Indianapolis, IN, USA) [70]. Briefly, 6-week old Tat transgenic mice were treated with 80 mg/kg of doxycycline in saline, injected intraperitoneally (IP) once daily for 0, 3, or 14 days. There were 4 mice per group for the 0 and 14 day groups, and 5 mice in the 3 day group. Mice were euthanized via cervical dislocation and brain tissue was immediately dissected at 4°C, flash frozen in isopentene, and stored at −80°C. RNA was isolated from half of each brain using the PureLink™ Total RNA Purification System (Invitrogen, Carlsbad, CA, USA) according to the manufacturer's protocol. Complementary DNA (cDNA) synthesis was performed using 2 µg total RNA, oligo-dT primers, and the Superscript III first-strand synthesis system (Invitrogen, Carlsbad, CA, USA). Gene-specific primer sequences were as follows: 1) Tat primers: forward 5′-GCATCCAGGGGATCAGCCTA-3′, reverse 5′-CTGATGAGCTCTTCGTCGCT-3′; 2) RelB primers: forward 5′-CCCCTACAATGCTGGCTCCCTGAA-3′, reverse 5′-CACGGCCCGCTCTCCTTGTTGATT-3′; 3) TNFα primers: forward 5′-ACTCCAGAACATCTTGGAAATAGC-3′, reverse 5′-GCGGATCATGCTTTCTGTGC-3′; 4) RelA primers: forward 5′-TCAAGATCAATGGCTACACAGG-3′, reverse 5′-GCATTCAAGTCATAGTCCCCG-3′; 5) GAPDH primers: forward 5′-TGATGACATCAAGAAGGTGGTGAA-3′, reverse 5′-TCCTTGGAGGCCATGTAGGCCAT-3′. Real-time RT-PCR was performed using iQ™ SYBR® Green PCR Supermix (Bio-Rad, Hercules, CA, USA) and 100nM gene-specific forward and reverse primers in 20 µL total volume. After denaturation for 3 min. at 95°C, the PCR was run for 40 cycles of 95°C for 30 sec., primer-specific melting temperature (T_m_ °C) for 1 min., and 72°C for 30 sec. using an iCycler instrument (Bio-Rad, Hercules, CA, USA). GAPDH served as an internal control in these experiments.

All animal experiments were carried out in accordance with the Animal Welfare Act and the National Institute of Health guidelines. The animal protocol was approved by the University Committee on Animal Resources of the University of Rochester Medical Center. The facilities and programs of the Vivarium and Division of Laboratory Animal Medicine of the School of Medicine and Dentistry are fully accredited by the Association for the Assessment and Accreditation of Laboratory Animal Care International (AAALAC) and are in compliance with state law, federal statute and NIH policy.

### Immunoprecipitation

Samples containing equal amounts of protein were pre-cleared by incubation with 2 µg of normal rabbit serum and 20 µL of a 50/50 protein A/G+ agarose beads solution (both from Santa Cruz Biotechnologies Inc., Santa Cruz, CA, USA) at 4°C on a rotator for 1h. After the pre-clear step, the beads were removed by centrifugation and samples were transferred to tubes with specific antibodies (2 µg per sample). Samples were incubated with the antibodies at 4°C for 18h on a rotator. 30 µL of 50/50 protein A/G+ agarose beads were added to the samples, then they were incubated with rotation for an additional 2h at 4°C. The beads were then washed extensively with ELB lysis buffer supplemented with 0.1mM PMSF, 1mM DTT, and a mixture of protease and phosphatase inhibitors, and then boiled for 5 minutes in 20 µL of Laemmli buffer. Immunocomplexes were separated using SDS-PAGE (7.5%), transferred to a nitrocellulose membrane and probed with the indicated antibodies.

### Statistical analysis

Mean data values and the standard error of the mean (SEM) were calculated for each variable. Data involving the analysis of multiple sample groups were analyzed by one-way ANOVA followed by Bonferroni's test for multiple comparisons. A value of *p*<0.05 was designated as statistically significant.

## Results

### Transgenic expression of Tat induces RelB synthesis in mouse brain

Tat transgenic (Tat-Tg) mice are used as a model of HIV-induced neurocognitive disorders, as they develop Tat-induced behavioral changes and neurological abnormalities including hunched posture, tremor, ataxia, slow motor movement, and seizures [70]. In addition, these mice exhibit neuropathologies including astrocytosis, degeneration of neuronal dendrites, neuronal apoptosis, and increased infiltration of activated monocytes [70], all of which replicate features of HIV-associated neurologic disease (HAND). These mice express a Dox-inducible Tat gene driven by the GFAP promoter, which drives Tat protein production by astrocytes in the CNS [70].

**Figure 1:**
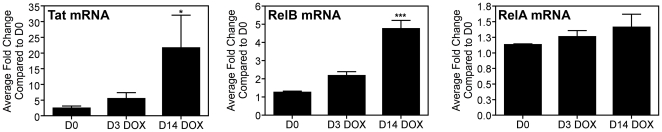
Transgenic expression of Tat induces RelB synthesis in mouse brain. 6-week old Tat-transgenic mice were induced with doxycycline for the indicated periods of time. Total RNA was extracted from brain tissue, reverse transcribed using oligo-dT primers, and subjected to Real-Time SYBR Green RT-PCR amplification. Fold induction of Tat, RelB and RelA mRNA species was normalized to GAPDH and presented as a function of the expression level in D0 samples. Data represent mean ± SEM of four replicates for D0 and D14 samples, and five replicates for D3 samples. Statistical significance (***, p<0.001 or *, p<0.05) is denoted as compared to D0 samples.

RelB was originally identified as a protein that controls inflammation [56], [57], [59], leading us to hypothesize that RelB may mediate Tat's effects in models of HAND. Thus, we first used quantitative real-time PCR (qRT-PCR) to determine whether Tat altered RelB or RelA expression levels in the brains of the Tat-Tg mice. Total RNA isolated from brain tissue of mice induced with doxycycline for 0, 3, or 14 days was used to measure the abundance of Tat, RelB, and RelA transcripts. As compared to non-induced Tat-Tg mice (Day 0, Dox–), there was an approximately 20-fold increase in Tat mRNA levels in 14 day Dox-treated animals (Figure 1). Since Tat is known to activate TNFα production, in order to confirm the activity of Tat, we also measured TNFα synthesis in Dox-exposed mice. Our results revealed an increase in TNFα mRNA in the brains of Dox-induced mice, validating the functional activity of Tat in these animals (data not shown). Consistent with these effects, we also observed a nearly 5-fold increase in RelB mRNA levels by day 14 Dox treatment, confirming that Tat also increased RelB expression (Figure 1). Interestingly, under these conditions, RelA mRNA levels remained unchanged, suggesting that the Tat-mediated increase in RelB was not a generalized effect of Tat on NF-κB subunits (Figure 1). Since total RNA was isolated from whole brain tissue, we could not determine which cell type most contributed to this Tat-induced increase in RelB message in this *in vivo* model. However, our further experiments in BV-2 microglial cells, primary human monocytes, and primary human astrocytes suggest that both microglia and astrocytes contribute to this increase.

### RelB inhibits TNFα and cytokine production triggered by Tat treatment

**Figure 2:**
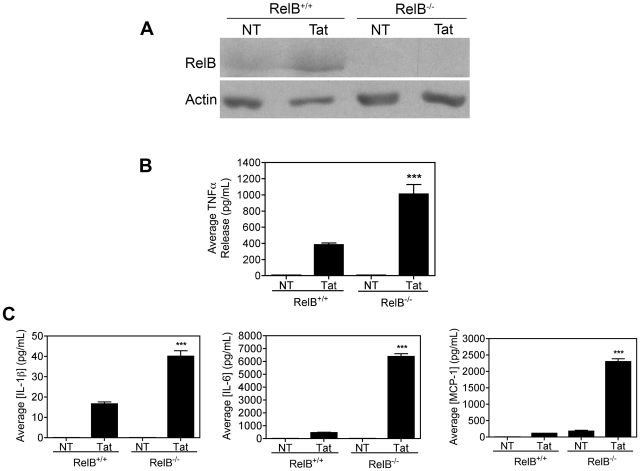
RelB inhibits Tat-induced TNFα and cytokine production in monocytes. *A,* Monocytes from RelB^+/+^ or RelB^−/−^ mice (3×10^5^) treated with Tat (100nM) for 8h and subjected to immunoblot analysis with either RelB-specific (*upper panel*) or Actin-specific (*lower panel*) antibodies, confirmed that Tat induced RelB in these cells. Data are representative of results from two separate experiments. *B*, Levels of TNFα in culture supernatants from these cells were analyzed by ELISA, and *C,* Levels of IL-1β, IL-6, and MCP-1 were anlayzed by Multi-Plex cytokine array as described in Results. Data is shown as mean ± SEM of values derived from three replicates each from two combined experiments. Statistical significance (p<0.001) is indicated (***), as compared with Tat treated RelB^+/+^ cells.

Microglia are believed to be principal mediators of HAND-associated neuropathology. Therefore, to explore the functional role of Tat-RelB induction in microglia, we examined Tat's ability to stimulate TNFα synthesis in magnetically sorted CD11b^+^ monocytic cells derived from the bone marrow of RelB deficient (RelB^−/−^) mice. We chose these cells to model microglia for three reasons: (1) microglia originate from myeloid lineage precursors in the bone marrow [71], (2) CD11b^+^ bone marrow cells are sensitive to Tat [72], and (3) highly homogeneous monocyte cultures can be obtained from the bone marrow of genetically modified mice [73]. The vast majority of CD11b-positive cells (nearly 95% of the total isolated population) were monocytes, as determined by morphologic criteria. Using immunoblot analyses, we first verified that 8h 100 nM Tat treatment stimulated RelB synthesis in these murine monocytes, and that RelB is indeed absent in RelB^−/−^ derived cells (Figure 2A). Again, following 8h 100 nM Tat treatment of these cells, we also collected culture supernatants and measured TNFα levels by ELISA. The results shown in Figure 2B reveal enhanced production of TNFα in Tat-treated RelB^−/−^ monocytes as compared to their RelB^+/+^ counterparts, strongly suggesting an anti-inflammatory role for RelB. The Bio-Plex Multi-Plex cytokine array assay was also used to measure additional inflammatory cytokine and chemokine levels in these same culture supernatants. Likewise, these results (Figure 2C) indicated enhanced production of IL-1β, IL-6, and MCP-1 in Tat-treated RelB^−/−^ monocytes compared to Tat-treated RelB^+/+^ cells, further supporting an anti-inflammatory role for RelB in the context of HIV. TNFα levels, as determined by ELISA above, were also validated using the Bio-Plex cytokine array, with similar results (data not shown).

**Figure 3:**
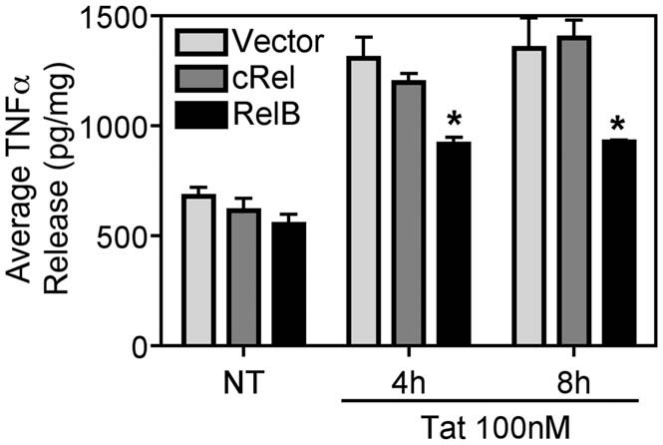
RelB inhibits Tat-induced TNFα production in BV-2 microglia. BV-2 cells (5×10^6^) were transiently transfected with either empty vector (pcDNA3.1-Myc/His-B), cRel, or RelB (10 µg), using Nucleofector (Amaxa/Lonza), and treated with Tat (100nM) for the indicated periods of time. TNFα release was measured by ELISA and normalized to total cellular protein content in the culture wells. Results are shown as mean ± SEM of values derived from two replicates from one representative experiment; two total experiments were performed. Statistical significance (p<0.05) is indicated, as compared to empty vector transfected cells (*).

We performed additional complementary experiments to test whether RelB overexpression in the microglial BV-2 cell line could reverse Tat's effects on TNFα synthesis. In contrast to cRel overexpression, which had no effect on Tat-induced TNFα production, RelB overexpression reduced Tat-induced TNFα release from BV-2 cells as we anticipated (Figure 3), further confirming the inhibitory properties of RelB.

Because RelB was over-expressed in these BV-2 cells, there is the possibility that over-expressing high levels of exogenous RelB could interfere with cells' ability to respond to Tat. This is not likely for two reasons: 1. cRel overexpression did not affect Tat's ability to induce TNFα production, and 2. Immunoblot analysis of transfected RelB levels, compared to RelB levels stimulated by 8h 100nM Tat treatment, showed no significant differences, thus suggesting that exogenous RelB levels resulting from transfection were similar to inducible RelB levels (data not shown). Together these data indicate that RelB's ability to reduce TNFα production in Tat-exposed cells results from a functional effect of RelB, and is not due to overexpression of RelB interfering with cells' ability to respond to Tat.

### Tat induces de novo synthesis of RelB

**Figure 4:**
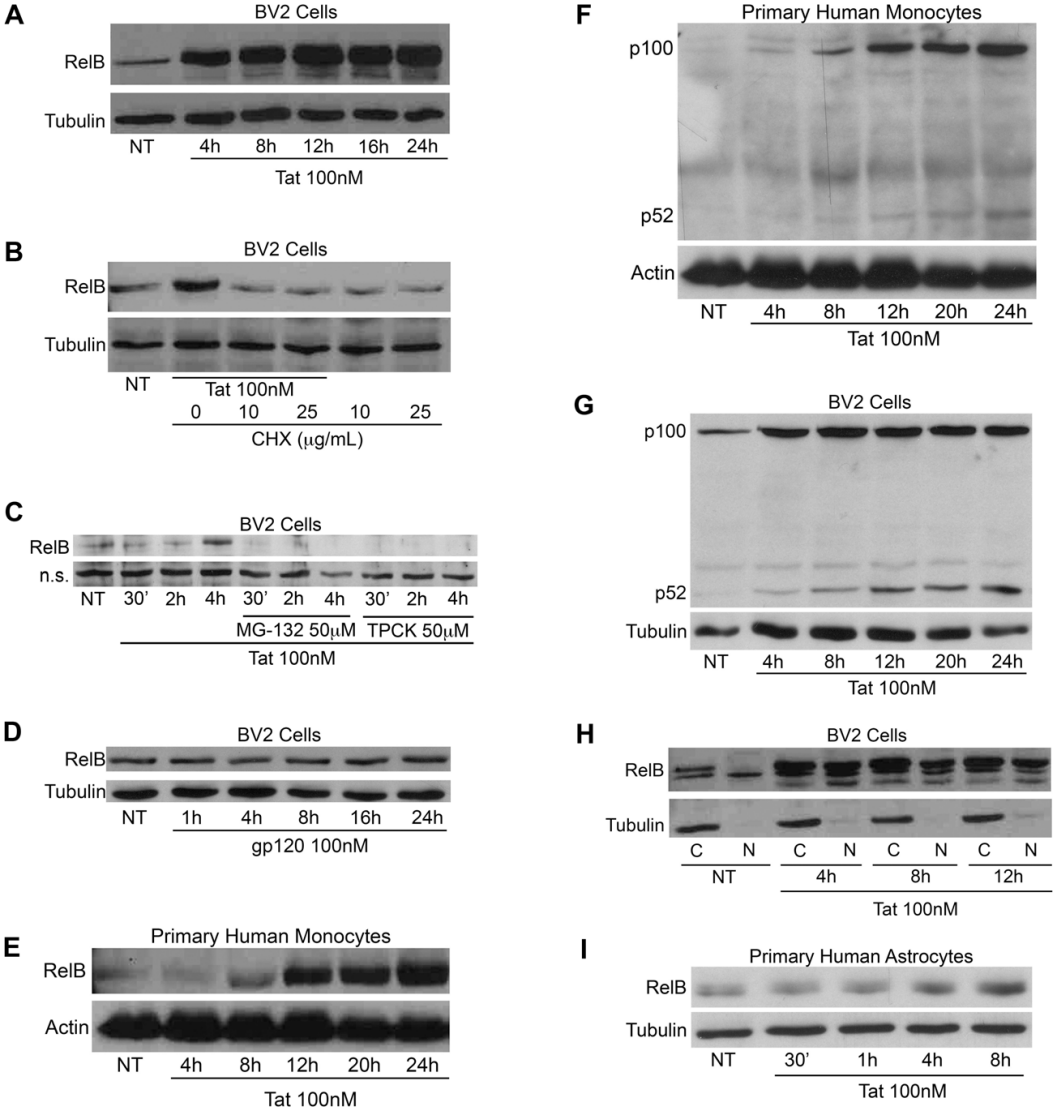
Tat induces de novo synthesis of RelB. *A,* BV-2 cells (1.2×10^5^) were treated with Tat (100nM) for the indicated periods of time and whole cell lysates were subjected to immunoblot analysis using either RelB-specific (*upper panel*) or α-Tubulin-specific (*lower panel*) antibodies. *B,* BV-2 cells (1.2×10^5^) were treated with Tat alone or together with CHX as indicated for 4h. Whole cell lysates were subjected to immunoblot analysis using either RelB-specific (*upper panel*) or α-Tubulin-specific (*lower panel*) antibodies. *C,* BV-2 cells (1.2×10^5^) were treated with Tat (100nM) alone or together with MG-132 or TPCK both at a concentration of 50µM for the indicated periods of time. Whole cell lysates were subjected to immunoblot analysis using a RelB-specific (*upper panel*) antibody. Levels of a nonspecific (n.s.) band (*lower panel*) are shown to indicate equal protein loading. *D,* BV-2 cells (1.2×10^5^) were treated with gp120 (SF162, 100nM) for the indicated periods of time and whole cell lysates were subjected to immunoblot analysis using either RelB-specific (*upper panel*) or α-Tubulin-specific (*lower panel*) antibodies. *E,* Primary human monocytes (2×10^5^) were treated with Tat for the indicated periods of time and whole cell lysates were subjected to immunoblot analysis using either RelB-specific (*upper panel*) or Actin-specific (*lower panel*) antibodies. *F,* Primary human monocytes (2×10^5^) were treated with Tat for the indicated periods of time and whole cell lysates were subjected to immunoblot analysis using either p100/p52-specific (*upper panel*) or Actin-specific (*lower panel*) antibodies. *G,* BV-2 cells (1.2×10^5^) were treated with Tat for the indicated periods of time and whole cell lysates were subjected to immunoblot analysis using either p100/p52-specific (*upper panel*) or α-Tubulin-specific (*lower panel*) antibodies. *H,* BV-2 cells (1.2×10^5^) were treated with Tat as indicated and cytosolic (labeled “C”) and nuclear (labeled “N”) extracts were subjected to immunoblot analysis with either RelB-specific (*upper panel*) or α-Tubulin-specific (*lower panel*) antibodies. *I,* Primary human astrocytes (4×10^4^) were plated 72h prior to treatment. These cells were treated with Tat as indicated and whole cell lysates were subjected to immunoblot analysis using either RelB-specific (*upper panel*) or α-Tubulin-specific (*lower panel*) antibodies.

Next we examined the mechanisms by which RelB regulates TNFα production through Tat. To do this we employed the easily genetically manipulatable microglial BV-2 cell line. First, we needed to confirm that the Tat-induced increases in RelB that we observed in our *in vivo* models, also translated to this *in vitro* model. Although activation of NF-κB involves cytosolic-nuclear redistribution of RelA (a major component of NF-κB) rather than *de novo* synthesis of this protein, NF-κB activation also positively regulates expression of other NF-κB family members, including RelB [74], [75]. Therefore, we speculated that Tat might be inducing *de novo* synthesis of RelB in our model systems. As shown in Figure 4A, relatively low levels of RelB were detected in untreated BV-2 cells. Within 4h, these levels were profoundly elevated by 100nM Tat and remained higher for up to 24h of Tat exposure. Under these conditions, Tubulin levels (protein loading control) were not altered. Further immunoblot analyses revealed that the Tat-induced increase in RelB expression resulted from enhanced RelB synthesis, as this effect was blocked by pre-treatment with the protein translation inhibitor Cycloheximide (CHX) (Figure 4B). Consistently, the Tat-induced increase in RelB expression was also blocked by pre-treatment of BV-2 cells with a proteasomal inhibitor, MG-132, or an inhibitor of chymotrypsin-like serine proteases, N-alpha-tosylphenylalanyl chloromethyl ketone (TPCK), suggesting that activation of the NF-κB signaling pathway might be crucial for this effect (Figure 4C). Furthermore, the increase in RelB levels was found to be a specific effect of Tat, as RelB was not increased by treatment with another HIV viral protein, gp120 (Figure 4D). However, gp120's failure to induce RelB synthesis was not due to failure of cellular activation by gp120, because, as expected [76], gp120 was able to induce TNFα release from BV-2 cells (data not shown).

Since microglia are a monocyte-derived cell type, we also examined RelB protein levels in primary human monocytes, to further translational relevance to primary human disease models. Analogous to the responses seen in BV-2 cells, treatment of primary human monocytes with Tat also resulted in increased RelB expression, albeit with delayed kinetics (Figure 4E), suggesting that Tat-mediated synthesis of RelB occurs in non-dividing cells as well. Active RelB is associated with NF-κB family member p52, which is proteolytically cleaved from the inhibitor p100 [48]. As such, further immunoblot analysis revealed increased p100 expression, and its processing into p52 molecules, in Tat-exposed primary human monocytes (Figure 4F), as well as in BV-2 cells (Figure 4G). Furthermore, we observed that this newly synthesized RelB rapidly relocates to the nucleus, and thus is functionally active (Figure 4H).

Astrocytes are the most abundant cell type in the brain, are non-productively infected with HIV-1 and, similar to microglia, can be activated by viral proteins such as Tat, thereby contributing to HIV-induced neuroinflammation [77]. For these reasons, we also measured RelB protein levels following Tat treatment of primary human astrocytes. As shown in Figure 4I, by 8h of Tat treatment there is increased RelB synthesis in astrocytes, while Tubulin levels remain unchanged.

### RelB inhibits TNFα synthesis at the transcriptional level, but RelA increases TNFα synthesis

**Figure 5:**
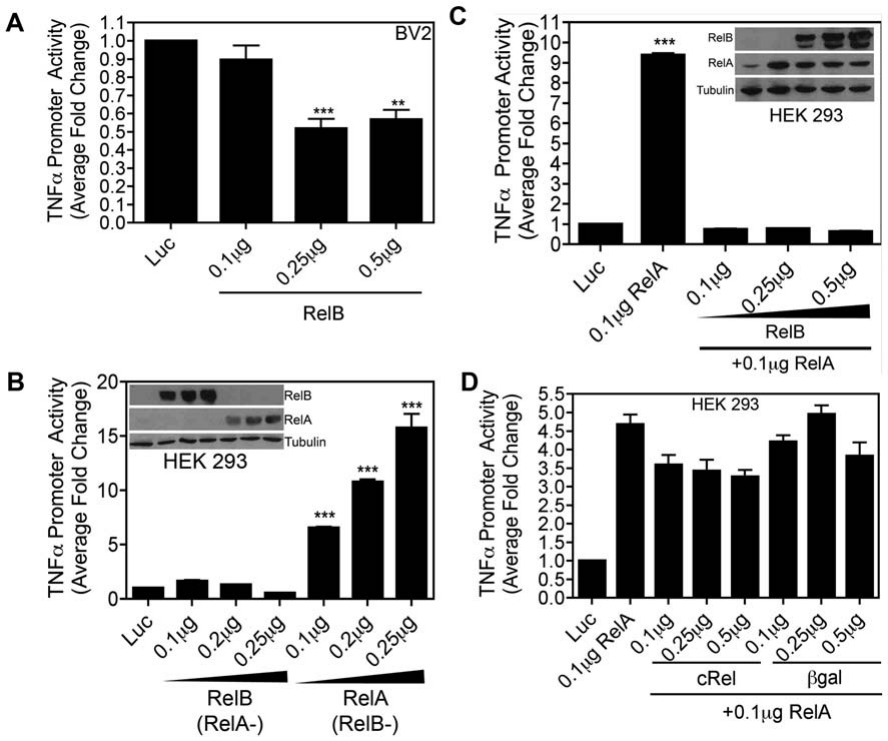
Opposing effects of RelA and RelB on TNFα promoter activity. *A,* BV-2 cells (1.2×10^5^) were transiently transfected with 0.25 µg of plasmid DNA containing a luciferase reporter gene under transcriptional control of the mouse TNFα promoter region in the absence or presence of increasing amounts of a RelB-encoding plasmid. 24h later cells were lysed and luciferase activity was determined. Total protein amount, as determined by Bradford assay was used to normalize the samples. Data are presented as fold change compared to cells transfected with the luciferase reporter alone. ***, p<0.001, and **, p<0.01 as compared to cells transfected with the luciferase reporter alone. *B,* HEK 293 cells (1.8×10^5^) were transiently transfected with the mouse TNFα promoter-luciferase reporter plasmid along with increasing amounts of a plasmid for RelA or RelB. The luciferase (i.e. TNFα promoter) activity in whole cell lysates was determined 24h post-transfection. Samples were subjected to immunoblot analysis using either RelA or RelB-specific antibodies to determine the expressed level of RelA and RelB protein. ***, p<0.001 as compared to cells transfected with the luciferase reporter alone. *C,* HEK 293 cells (1.8×10^5^) were transiently transfected with the mouse TNFα promoter-luciferase reporter plasmid along with a plasmid for RelA in the absence or presence of increasing amounts of RelB-encoding plasmid. The luciferase activity in whole cell lysates was determined 24h post-transfection. Samples were subjected to immunoblot analysis using either RelA or RelB-specific antibodies to determine the expressed level of RelA and RelB protein. ***, p<0.001 as compared to cells transfected with the luciferase reporter alone. *D,* HEK 293 cells (1.8×10^5^) were transiently transfected with the mouse TNFα promoter-luciferase reporter along with a plasmid for RelA in the absence or presence of increasing amounts of cRel or β-galactosidase-encoding plasmid. Luciferase activity in whole cell lysates was determined 24h post-transfection.

To better understand how RelB inhibits Tat-induced TNFα production, we used a reporter gene assay. To do this, BV-2 cells were transiently transfected with a TNFα-promoter luciferase reporter plasmid, either alone or together with increasing amounts of WT RelB plasmid. The total amount of DNA in each transfection was kept constant by addition of an appropriate amount of pcDNA3.1 empty vector plasmid. These results revealed that basal TNFα promoter activity was blocked by overexpression of RelB (Figure 5A). To compare the effects of RelA overexpression on TNFα promoter activity, we performed similar additional luciferase assays in which HEK 293 cells were transfected with the TNFα-promoter luciferase reporter, together with increasing amounts of either RelA or RelB. As shown in Figure 5B, in contrast to RelB, overexpression of increasing amounts of RelA led to dose-dependent *activation* of the TNFα promoter. These latter studies utilized HEK 293 cells for their high transfection yields, and their robust (and thus readily detectable) increases in TNFα promotor activity with RelA expression, thus allowing us to determine the effects of RelB expression on RelA transcriptional activity.

### RelB interaction with RelA inhibits upregulation of the TNFα promoter by RelA

Previous reports have shown that RelB and RelA interact, and that this interaction can lead to inhibition of either RelA-dependent or RelB-dependent gene expression in multiple cell types [52]–[55]. Based on these reports, first we examined the effect of RelB overexpression on RelA-induced activation of the TNFα promoter. Overexpression of RelA alone resulted in an approximately 10-fold increase in TNFα promoter activation, an effect that was completely blocked by expressing increasing amounts of RelB (Figure 5C). Interestingly, consistent with Figure 3, overexpressing increasing amounts of cRel (relevant control), or β-galactosidase (irrelevant control), did not significantly inhibit RelA-dependent activation of the TNFα promoter (Figure 5D), suggesting that RelB exerts a selective inhibitory effect on RelA transcriptional activity.

**Figure 6:**
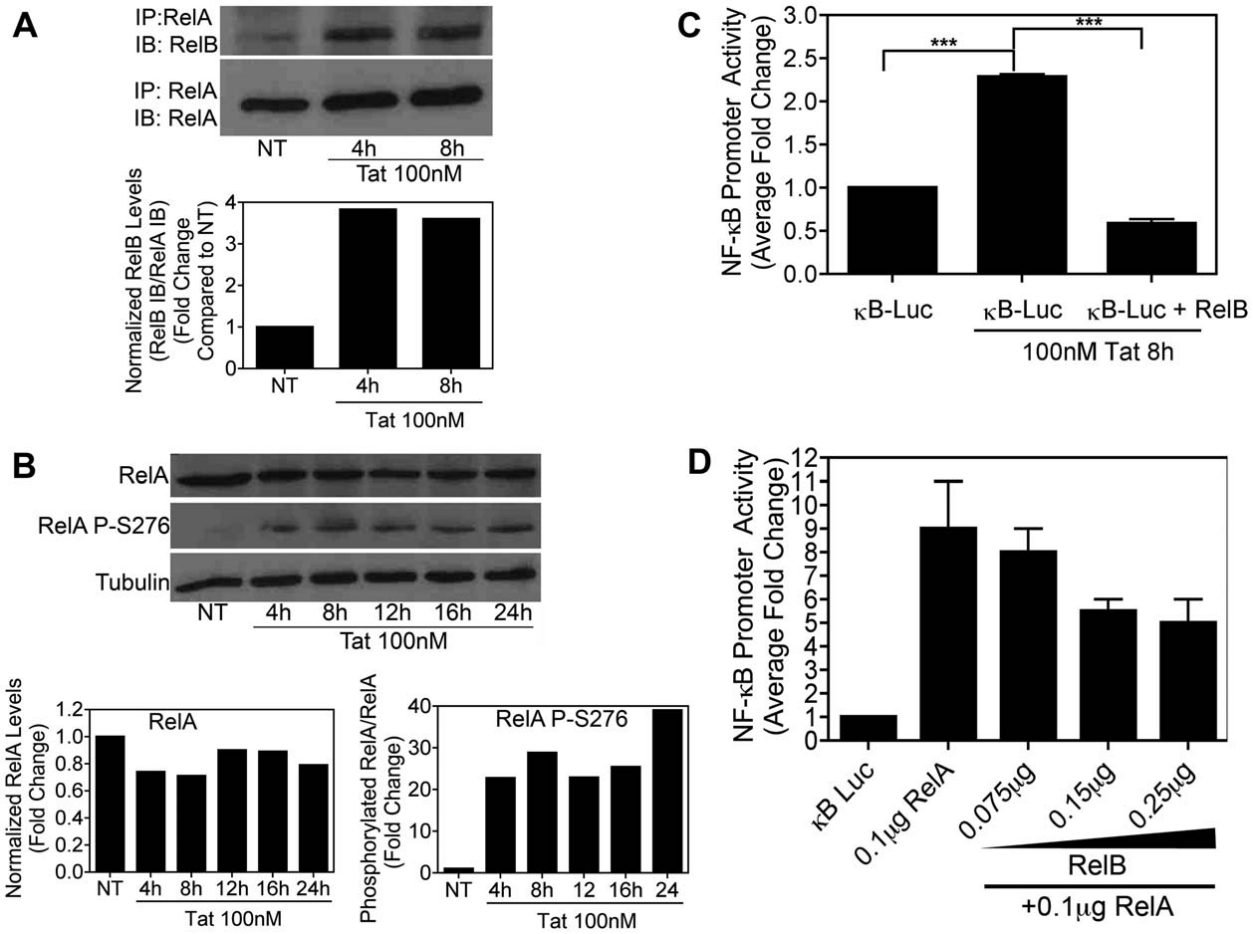
RelB inhibits NF-κB activation via physical interaction with RelA. *A,* BV-2 cells (8×10^5^) were treated with Tat (100nM) for the indicated periods of time. Whole cell lysates were subjected to immunoprecipitation using a RelA-specific antibody and Protein A/G+ agarose beads. Immunocomplexes were separated by 7.5% SDS-PAGE and blotted onto nitrocellulose membrane and subjected to immunoblot analysis with antibodies specific for RelB or RelA. The plot below the bands represents densitometry values for each band, normalized as a ratio of RelA(IP)/RelB(IB) (i.e. top bands): RelA(IP)/RelA(IB) (i.e. bottom bands), to indicate the increase in RelB/RelA interactions at each time point. *B,* BV-2 cells (1.2×10^5^) were treated with Tat (100nM) for the indicated periods of time and whole cell lysates were subjected to immunoblot analysis using either RelA-specific (*upper panel*), RelA phospho-serine 276-specific (*center panel*) or α-Tubulin-specific (*lower panel*) antibodies. Protein levels were quantified using ImageJ software (bottom graphs). The results of a single representative experiment are shown. *C,* BV-2 cells (5×10^6^) were transiently transfected using Nucleofector (Amaxa/Lonza) with an NF-κB-dependent luciferase reporter plasmid either alone or together with a RelB-encoding plasmid. 16h post-transfection cells were either left untreated or were treated with Tat (100nM) for 8h. Luciferase activity in whole cell lysates was determined. Results are shown as mean ± SEM of values derived from three replicates from one representative experiment; two total experiments were performed. Statistical significance (p<0.001) is indicated (***). *D,* BV-2 cells (1.5×10^5^) were transiently transfected using Lipofectamine (Invitrogen) with the NF-κB-luciferase reporter plasmid together with a plasmid for RelA in the absence or presence of increasing amounts of RelB-encoding plasmid. The luciferase activity in whole cell lysates was determined 18h post-transfection. Results are shown as mean ± SEM of values derived from two replicates from one representative experiment; two total experiments were performed.

Next, to determine whether RelB interacts with RelA to achieve these effects, we tested whether Tat stimulates protein-protein interactions between endogenous RelA and RelB in BV-2 cells. After addition of Tat for either 4h or 8h, whole cell lysates of BV-2 cells were collected, and RelA protein complexes were captured with a RelA-specific antibody using Protein A/G+ agarose beads. The bound proteins were then visualized by immunobloting with a RelB-specific antibody. By 4h of Tat treatment there was a significant increase in RelB protein that was pulled down with the RelA antibody, thus demonstrating that Tat treatment was stimulating interactions between RelA and RelB in BV-2 cells (Figure 6A, top panel). The blot was stripped and re-probed with a RelA-specific antibody to confirm that approximately equivalent amounts of RelA protein were pulled down in each sample (Figure 6A, bottom panel). Comparative densitometric analysis of these bands, normalized to the RelA IP/RelA IB bands (bottom panel), informed us that Tat increased RelB/RelA interactions by 4-fold at both 4h and 8h (Figure 6A, bar graph).

It was previously reported that RelA phosphorylation at serine 276 is required for the interaction between RelB and RelA to occur [53]. Accordingly, using an antibody that specifically recognizes serine 276 phosphorylated RelA, we were able to confirm that RelA is indeed phosphorylated at serine 276 in Tat-treated BV-2 cells (Figure 6B, blots). Quantification of these blots found a 20–40 fold increase in the phosphorylated form of RelA in Tat treated BV-2 cells versus controls (Figure 6B, graphs).

Thus, we predicted that this Tat-induced interaction between RelB and RelA would therefore inhibit Tat-induced, RelA-mediated transcriptional activation of NF-κB pathways. Indeed, using an NF-κB luciferase reporter plasmid in BV-2 cells, our results confirmed that overexpression of RelB inhibits activation of endogenous NF-κB induced by 8h 100 nM Tat treatment (Figure 6C). Likewise, analogous to the results shown in Figure 5C, we observed a dose-dependent inhibition of RelA-mediated NF-κB luciferase transcription following overexpression of RelB in BV-2 cells (Figure 6D).

### The entire Rel Homology Domain of RelB is necessary for the interaction with RelA

**Figure 7:**
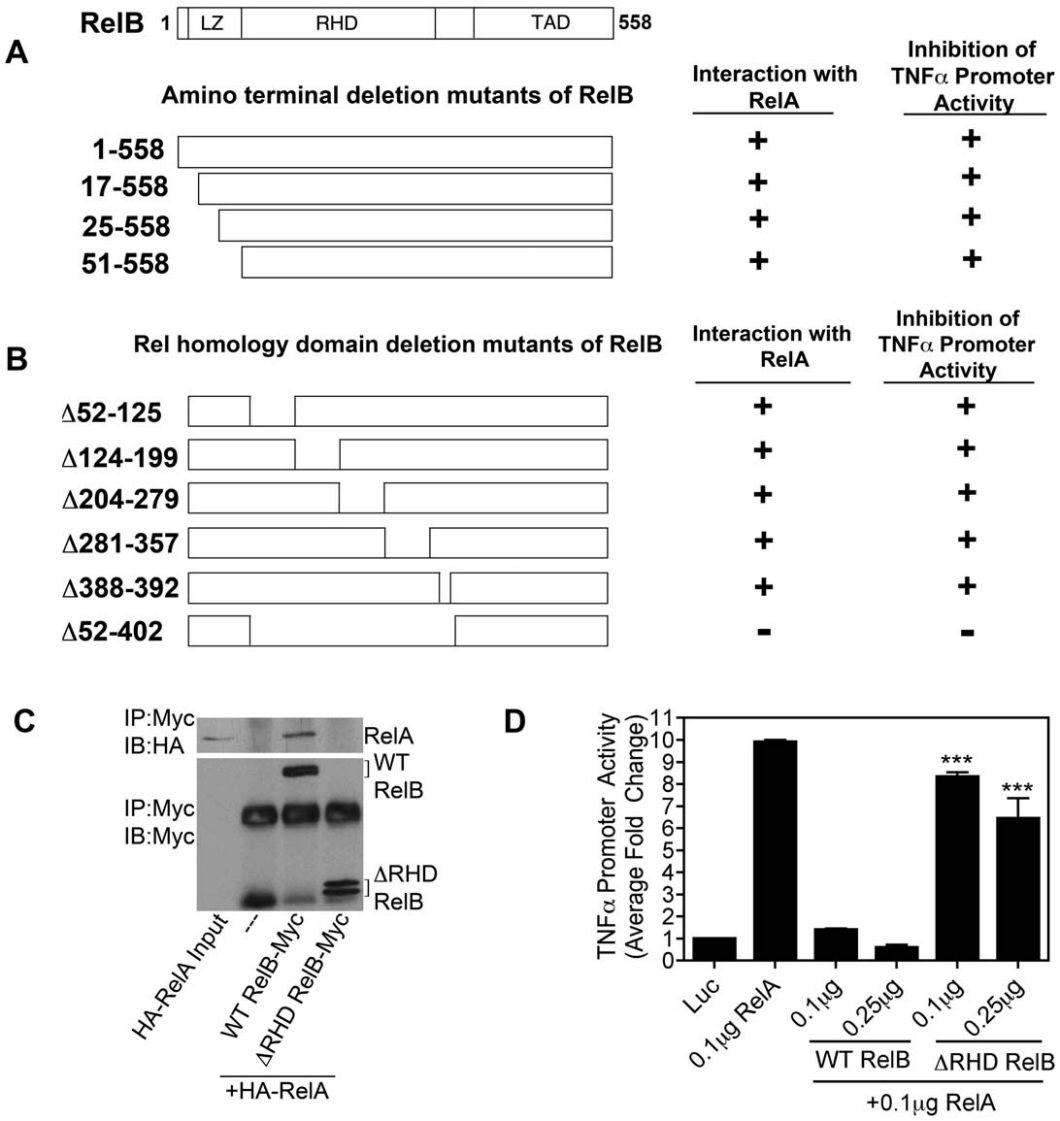
RelB Rel homology domain required for interaction with RelA and blockade of TNFα promoter activity. *A, B,* Diagrams show representation of full length and deletion mutants of RelB used to determine the domains of RelB necessary for interaction with RelA. HEK 293 cells (1.8×10^5^) were transiently transfected with either HA-tagged RelA or Myc-tagged RelB deletion mutants alone or in combination. Interaction was determined by immunoprecipitation with Myc-specific antibody and Protein A/G+ agarose beads followed by immunoblot analysis with HA-specific or Myc-specific antibodies. Interacting (+) and non-interacting (−) mutants are indicated. These RelB deletion mutants were also analyzed using the TNFα promoter-luciferase reporter plasmid co-transfected into HEK 293 cells together with a plasmid for RelA in the absence or presence of increasing amounts of RelB-encoding plasmid (WT or deletion mutants). Inhibition of RelA activation of the TNFα promoter is indicated (+/−). *C,* HEK 293 cells (1.8×10^5^) were transiently transfected with either HA-tagged RelA or Myc-tagged RelB Δ52–402 alone or in combination. Interaction was determined by immunoprecipitation with Myc-specific antibody and Protein A/G+ agarose beads followed by immunoblot analysis with HA-specific or Myc-specific antibodies. *D,* RelB Δ52–402 was also analyzed using the TNFα promoter-luciferase reporter plasmid co-transfected into HEK 293 cells along with a plasmid for RelA in the absence or presence of increasing amounts of RelB Δ52–402-encoding plasmid. Statistical significance (p<0.001) as compared to cells transfected with 0.1 µg WT RelB+RelA is indicated (***).

To clarify how RelB might interact with RelA to inhibit activation of the TNFα promoter, sequential deletion mutants within RelB were made. These RelB deletion mutants were overexpressed in HEK 293 cells together with HA-tagged RelA. Co-immunoprecipitation (co-IP) was then used to test for interactions between these proteins. Parallel cultures were also co-transfected with the TNFα-promoter luciferase reporter, to determine which of these deletion mutated RelB derivatives failed to block transcriptional activity of RelA, which would suggest an inhibitory domain interaction with RelA. We first focused on the amino terminal leucine zipper domain of RelB, because this domain is unique to RelB among the NF-κB family members. Leucine zipper domains are also known to be involved with protein-protein interactions [46]. However, amino terminal truncation mutants of RelB, including deletion of the entire leucine zipper domain, still interacted with HA-RelA as shown by co-IP, and these mutants also inhibited RelA activity equally well (Figure 7A), suggesting that this region was not responsible for the transcription-inhibiting interactions of RelB on RelA.

We then introduced sequential deletions within the Rel Homology Domain (RHD) of RelB (Figure 7B). The RHD of NF-κB proteins is known to be responsible for homo- and hetero-dimer formation with other NF-κB proteins [78]. All of these sequential RelB RHD deletion mutants, as well as deletion of the proximal nuclear localization signal located between residues 388–392 within RelB, still formed complexes with RelA and inhibited its activity (Figure 7B). However, the ability of RelB to interact with RelA, and to inhibit RelA-dependent activation of the TNFα promoter, was lost following deletion of the entire RHD (Δ52–402, Figure 7B; aka ΔRHD RelB, Figures 7C and 7D). This is reported in tabular format in Figure 7B (bottom line), with Figures 7C and 7D illustrating the failure of ΔRHD RelB to interact with RelA (7C), and to inhibit RelA activation of TNFα transcriptional activity (7D), respectively. These results indicate that the entire RHD of RelB is necessary for its interaction with RelA.

### Amino terminal region of Tat is necessary for RelB induction in microglia

**Figure 8:**
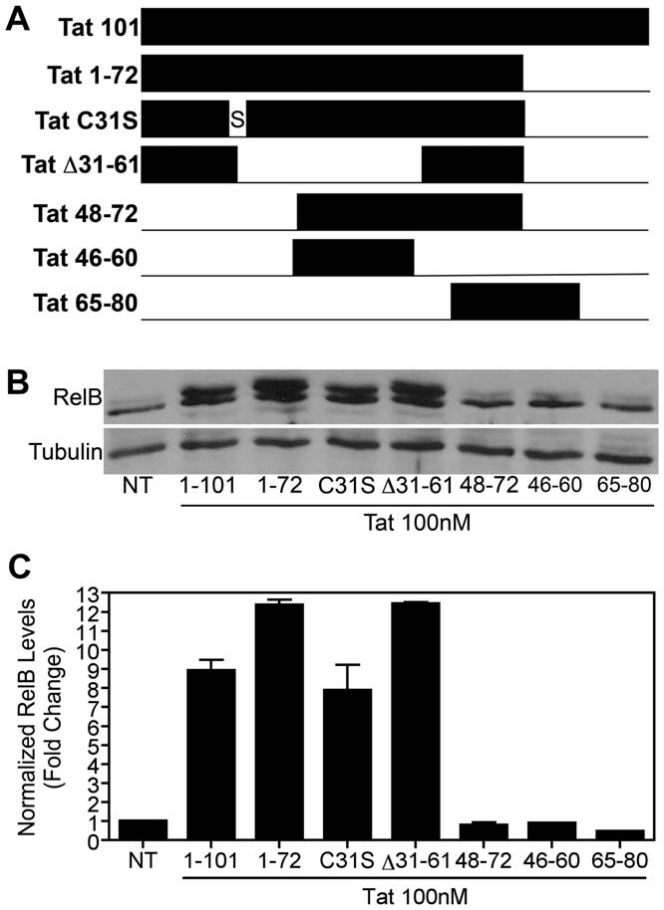
Amino terminal region of Tat is necessary for RelB induction in microglia. *A,* Diagram shows a representation of full length, mutated and truncated Tat proteins and peptides used in these experiments. *B,* BV-2 cells (1.2×10^5^) were treated with Tat (100nM) for 8h and whole cell lysates were subjected to immunoblot analysis using either RelB-specific (*upper panel*) or α-Tubulin-specific (*lower panel*) antibodies. These results indicate that standard Tat 1–101, Tat 1–72, Tat C31S, and Tat Δ31–61 all activated RelB, whereas Tat peptides 48–72, 46–60 and 65–80 did not. *C,* Densitometry quantification of immunoblots shown in *B*. RelB levels were normalized to Tubulin levels and fold change compared to non-treated (NT) was calculated.

The HIV-1 protein Tat is a multi-functional protein. Residues 1–72 are encoded by the first exon and residues 73–101 are encoded by a second exon [79]. It is released from infected microglia, macrophage and astrocytes in the CNS [80]. Despite its small size, Tat has multiple domains conferring different functional effects. For example, amino acids 1–48 contain protine-rich as well as cysteine-rich regions that represent a minimal activation domain of HIV-1 Tat required for activation of the HIV-1 promoter region, known as the long terminal repeat (LTR). The basic domain 49–72 contains an RKKRRQRRR motif, which confers the RNA binding activity of Tat and is also important for cellular uptake and nuclear distribution of this protein [81], while amino acids 65–80 of Tat include the RGD motif that is involved in binding to cellular integrins [81], [82]. Sequences from 22–37, a cysteine-rich domain, bind with divalent cations like zinc and cadmium, thereby inducing the dimerization and subsequent inactivation of Tat [83]. Mutations in the region 1–21 are tolerant to changes without the loss of biologic activity; in contrast, changes in amino acids 25–40 are generally deleterious for transactivation. Moreover, substitution at cysteine residues 22, 25, 27 and 37 alters transactivation of the HIV LTR [81]. The cysteine residue at position 31 is critical for binding to the NMDA receptor on neurons and mediating neurotoxicity [84]. Chemotactic properties have also been attributed to this residue [85]. Here we have tested various length Tat proteins to determine which domains are necessary for induction of RelB synthesis in microglial cells. A diagram depicting the Tat peptides we used to treat BV-2 cells is shown in Figure 8A.

As shown in Figure 8, full-length Tat (Tat 1–101) and Tat 1–72 both induced an approximately 10–12-fold increase in RelB levels by 8h of treatment in BV-2 cells, suggesting that the first exon of Tat is required for RelB induction in microglia. Similarly, Tat C31S and Tat Δ31–61 treatments led to a 10–12-fold increase in RelB synthesis, suggesting that the altered residues in these variants are dispensible. In contrast, Tat 48–72, 46–60 and 65–80 did not induce RelB synthesis in BV-2 cells, indicating that the basic motif within Tat is not sufficient to stimulate RelB production. Our results suggest that the amino terminal region, consisting of amino acids 1–30 of Tat, is critical for Tat-dependent RelB activation in microglia. It may also be that multiple domains within the first exon of Tat are necessary for the Tat-induced increase in RelB synthesis. This possibility is currently under investigation in our laboratory.

## Discussion

Neuroinflammatory responses and monocyte/macrophage CNS infiltration appear critical to the development of HAND even with cART, and are not significantly reduced by cART [86], [87], thus, this remains a critical area of investigation in the pursuit of adjunctive therapies for reducing the impact of HIV on the nervous system. We, and colleagues, have previously shown that TNFα released from HIV infected or activated monocytes/microglia is a major contributor to HIV-induced neuroinflammation that leads to neuron damage and cognitive impairment [45], [88]–[94]. Moreover, many key aspects of TNFα signaling occur via NF-κB [95], [96], and NF-κB signaling is likely to be a key mediator of TNFα's neurotoxic actions in HAND, particularly in regards to activation and HIV infection of brain macrophages and microglia [97], [98]. Accordingly, TNFα and NF-κB signal transduction are principal mechanistic pathways by which Tat and other viral proteins or mediators are thought to perpetuate HIV-induced nervous system damage [99]–[108]. RelB, in turn, is an NF-κB family molecule with known anti-inflammatory properties and modulatory roles in NF-κB signal transduction. While Rel family members have previously been implicated as possible mediators of HIV- and Tat-dependent transcription [100], the precise mechanisms of this regulation remain unclear. Furthermore, RelB modulation of cytokine and TNFα activation in microglial cells is an area that may have significant importance for understanding HAND pathology, yet until now has remained unexplored.

Herein we describe a novel inhibitory regulatory feedback loop for reducing inflammatory responses in microglial cells, whereby RelB protein-protein interactions with RelA inhibit RelA's transcriptional activity, to regulate NF-κB mediated cytokine synthesis. Early reports indicated that RelB exclusively formed heterodimers with p50 and p52. It was not until 2003 that Marienfeld et. al reported an interaction between RelB and RelA in multiple cell types [52]. RelB/RelA interactions may not have been reported earlier because this complex might not bind well to typical κB DNA binding elements, or the level of this complex might be low compared to levels of other RelB complexes. Additional reports of an inhibitory RelB/RelA interaction have since been described in fibroblasts, dendritic cells, and THP-1 human monocyte cells [53]–[55].

Here we describe for the first time, direct inhibition of RelA-dependent TNFα transcription by RelB in microglia, as a result of RelB/RelA protein interactions. These findings are supported by other work showing that LPS stimulation of RelB^−/−^ fibroblasts resulted in enhanced production of cytokines IL-1α, IL-1β, and TNFα [58], and chemokines RANTES, MIP-1α, MIP-1β, MIP-2, IP-10, JE/MCP-1, and KC/CINC [59]. In addition, another study demonstrated that RelB expression correlated with IL-1β repression in human monocyte THP-1 cells [55]. All these findings likely have parallels with our findings herein, that RelB deficiency leads to enhanced Tat-induced TNFα transcriptional activity, and that RelB expression reduces this activity. On the other hand, and in stark contrast, macrophages isolated from RelB^−/−^ deficient mice demonstrated impaired TNFα production in response to LPS [57]. This study emphasizes that the exact consequences of RelB regulation of TNFα may be specifically dependent upon both the nature of the inflammatory insult, and upon the cell type affected – as is commonly the case with immunomodulatory signal transduction – thus highlighting the need for further studies of these effects in other cell types relevant to HIV neurologic disease, as we seek to further validate RelB as a potential therapeutic target in HAND.

Finally, since TNFα can be a downstream modulator of Tat's effects [104], it is possible that TNFα, rather than Tat directly, is the molecule responsible for inducing the RelA serine 276 phosphorylation that we observed in Tat-treated microglia. In addition, it is likely that either Tat- or TNFα-induced activation of NF-κB could also explain the increase in RelB, as RelB is known to be induced by NF-κB, and blockade of NF-κB signaling mechanisms with inhibitors MG-132 or TPCK inhibited the increase in microglial RelB. While additional studies are needed to further clarify these mechanisms, these possibilities do not conflict with our supposition that the RelB/RelA interactions shown here in Tat-treated microglial cells, represent a novel negative regulatory feedback loop for the resolution of inflammation in microglial cells, specifically by inhibiting RelA activation of pro-inflammatory cytokines such as TNFα. This mechanism may explain why more severe cognitive impairment is not present until late in the course of HIV infection, with HIV-induced inflammatory mediators provoking a constant “yin and yang” between inflammation and resolution of inflammation in the CNS.

Uncovering new mechanisms involved in resolving CNS inflammation has many potential benefits both in terms of reducing inflammation-induced neuronal damage, and also possibly limiting viral spread by reducing inflammation-induced influx of monocytes into the CNS. Moreover, TNFα and related cytokines have pleiotropic neuromodulatory, neurotoxic, and even neuroprotective functions [109], and are implicated in a wide range of CNS and peripheral disease states. Thus, identifying this novel negative regulatory feedback loop involving RelB could be particularly useful as an adjunctive therapeutic approach for HAND, peripheral HIV infection, or even other neurologic or immune diseases, since overexpression of RelB in microglial cells does not completely block Tat-induced release of TNFα, which would negatively impact TNFα's other endogenous functions. In total, these results described herein represent an important first-step toward furthering our understanding of RelB as a potentially important mediator of wide-ranging disease states.

## Supporting Information

Text S1 Recombinant Tat produced in eukaryotic cells activates microglia.
(0.04 MB DOC)

Figure S1 Recombinant Tat produced in eukaryotic cells activates microglia. A, HIV-1 Tat 1–72 protein was purified from recombinant baculovirus infected Sf9 insect cell cultures. Increasing volumes of the eluted protein were subjected to immunoblot analysis using antiserum to HIV-1 Tat. The Tat-specific band is indicated. B, BV-2 cells (1.2×10^5^) were treated with Sf9-Tat (1–72, 100nM), or E.Coli-Tat (1–72, 100nM, from Dr. A. Nath) for 8h and levels of TNFα in the culture supernatant quantified by ELISA. Data correspond to mean ± SEM of three replicates, from a single representative experiment; the experiment was performed twice. C, BV-2 cells (1.2×10^5^) were treated with Sf9-Tat (100nM) for the indicated periods of time and whole cell lysates were subjected to immunoblot analysis using either IκBα-specific (upper panel) or α-Tubulin-specific (lower panel) antibodies. Data are representative of results from two separate experiments. D, For the EMSA, 4 µL of nuclear protein extract from BV-2 cells treated with Sf9-Tat (100nM) for the indicated times was incubated together with a ^32^P-labeled palindromic NF-κB oligonucleotide. The samples were separated on a native polyacrylamide gel that was then fixed, dried, and exposed to x-ray film. The two major DNA binding complexes consisting of NF-κB family members, as labeled, were identified by antibody supershift analysis.
(0.67 MB TIF)
