# Supplementary material for: Nuclear Factor-Kappa B Family Member RelB Inhibits Human Immunodeficiency Virus-1 Tat-Induced Tumor Necrosis Factor-Alpha Production
Source: PLoS One. 2010 Jul 29;5(7):e11875. doi: 10.1371/journal.pone.0011875 (PMC2912378; doi:10.1371/journal.pone.0011875)
Supplement: Text S1 — Recombinant Tat produced in eukaryotic cells activates microglia. (0.04 MB DOC) [file pone.0011875.s001.doc]

**Supplementary Text S1**

*Recombinant Tat produced in eukaryotic cells activates microglia*

Following infection of human cells, immediate-early gene products of HIV-1 undergo multiple post-translational modifications. For example, Tat is often modified by phosphorylation, acetylation, ubiquitination, and methylation at specific residues, through which various functions of this protein are regulated [1-3]. Since numerous signaling pathways leading to protein modification in human cells are also shared by insect cells, we produced the HIV-1 Tat gene product in Fall armyworm *Spodoptera frugiperda* (Sf9) cells using a baculovirus expression system, thus allowing the production of recombinant protein that is functionally similar to a native mammalian protein. By using polyclonal antibodies against Tat (clone number 705, obtained from the NIH AIDS Research and Reference Reagent Program), we performed immunoblot analyses which revealed that the purified protein is indeed antigenically similar to native Tat (Figure S1A).

Secondly, to test whether eukaryotic post-translational modifications are necessary for Tat to optimally activate microglia, BV-2 cells were treated with recombinant Tat 1-72 that was produced in either Sf9 cells (Sf9-Tat), or in E. coli. (E. coli-Tat; kind gift of Dr. Avi Nath), followed by measurement of TNFα levels in the culture supernatants by ELISA. As shown in Figure S1B, the extent of TNFα production in response to Sf9-Tat or E. coli-Tat was comparable, suggesting that eukaryotic post-translational modifications are not required for microglial production of TNFα induced by Tat. We further verified that treatment of BV-2 cells with vehicle alone did not result in significant TNFα production (data not shown).

Finally, we examined whether Sf9-Tat is capable of inducing proteolysis of IκBα molecules and subsequent activation of NF-κB in microglial cells, as these events are normally observed in microglial cells activated by E. coli-Tat [4,5]. Indeed, high levels of IκBα were detected in whole cell lysates from untreated BV-2 cells (Figure S1C), and as expected incubation of these cells with Sf9-Tat led to the rapid degradation of IκBα, followed by re-synthesis of this molecule. Under these conditions, no change was observed in Tubulin protein levels. Activation of NF-κB was then investigated by performing electrophoresis mobility shift assays (EMSA) in which nuclear extracts isolated from Sf9-Tat-treated BV-2 cells were incubated with a double stranded radiolabeled oligonucleotide probe containing a high affinity κB site. As shown in Figure S1D, EMSA revealed the appearance of two major DNA binding complexes in treated cells, which were identified by antibody super-shift analyses as complexes consisting of NF-κB family members. As expected [5], DNA binding activity of the Oct-1 transcription factor was unaltered following cellular exposure to Sf9-Tat. Taken together, our results indicate that Sf9-Tat (referred to as ‘Tat’) activates microglial NF-κB pathways comparably to E. coli-Tat that is commonly used in the field, and that eukaryotic post-translational modifications within Tat may not be necessary for this microglial activation.

**References**

1. D'Orso I, Frankel AD (2009) Tat acetylation modulates assembly of a viral-host RNA-protein transcription complex. Proc Natl Acad Sci U S A 106: 3101-3106.

2. Van Duyne R, Easley R, Wu W, Berro R, Pedati C, et al. (2008) Lysine methylation of HIV-1 Tat regulates transcriptional activity of the viral LTR. Retrovirology 5: 40.

3. Hetzer C, Dormeyer W, Schnolzer M, Ott M (2005) Decoding Tat: the biology of HIV Tat posttranslational modifications. Microbes Infect 7: 1364-1369.

4. Nicolini A, Ajmone-Cat MA, Bernardo A, Levi G, Minghetti L (2001) Human immunodeficiency virus type-1 Tat protein induces nuclear factor (NF)-kappaB activation and oxidative stress in microglial cultures by independent mechanisms. J Neurochem 79: 713-716.

5. Sui Z, Sniderhan LF, Schifitto G, Phipps RP, Gelbard HA, et al. (2007) Functional synergy between CD40 ligand and HIV-1 Tat contributes to inflammation: implications in HIV type 1 dementia. J Immunol 178: 3226-3236.
