# Supplementary figures and images for: Nuclear Factor-Kappa B Family Member RelB Inhibits Human Immunodeficiency Virus-1 Tat-Induced Tumor Necrosis Factor-Alpha Production
Source: PLoS One. 2010 Jul 29;5(7):e11875. doi: 10.1371/journal.pone.0011875 (PMC2912378; doi:10.1371/journal.pone.0011875)

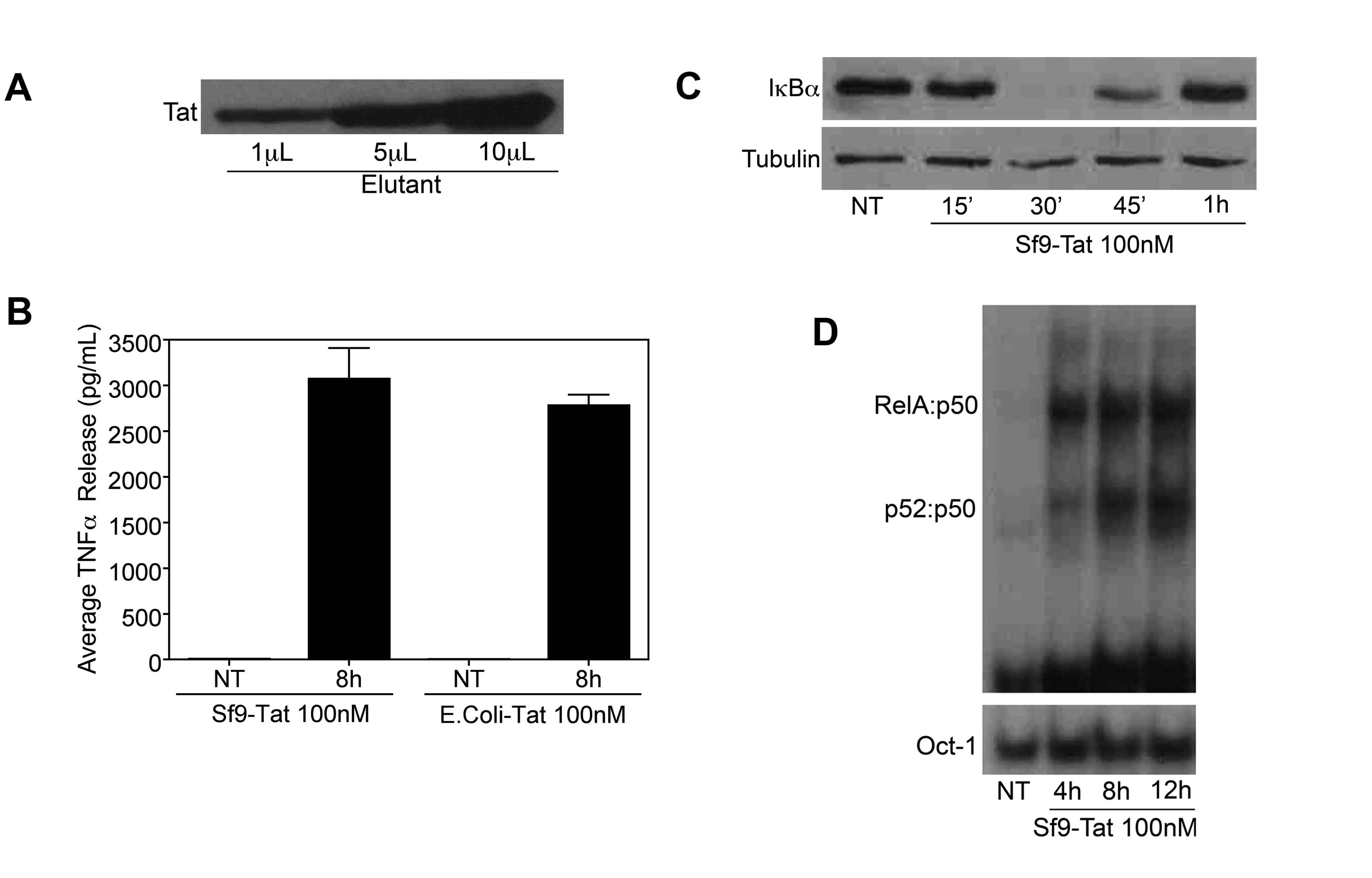

Supplement: Figure S1 — Recombinant Tat produced in eukaryotic cells activates microglia. A, HIV-1 Tat 1–72 protein was purified from recombinant baculovirus infected Sf9 insect cell cultures. Increasing volumes of the eluted protein were subjected to immunoblot analysis using antiserum to HIV-1 Tat. The Tat-specific band is indicated. B, BV-2 cells (1.2×10^5^) were treated with Sf9-Tat (1–72, 100nM), or E.Coli-Tat (1–72, 100nM, from Dr. A. Nath) for 8h and levels of TNFα in the culture supernatant quantified by ELISA. Data correspond to mean ± SEM of three replicates, from a single representative experiment; the experiment was performed twice. C, BV-2 cells (1.2×10^5^) were treated with Sf9-Tat (100nM) for the indicated periods of time and whole cell lysates were subjected to immunoblot analysis using either IκBα-specific (upper panel) or α-Tubulin-specific (lower panel) antibodies. Data are representative of results from two separate experiments. D, For the EMSA, 4 µL of nuclear protein extract from BV-2 cells treated with Sf9-Tat (100nM) for the indicated times was incubated together with a 32P-labeled palindromic NF-κB oligonucleotide. The samples were separated on a native polyacrylamide gel that was then fixed, dried, and exposed to x-ray film. The two major DNA binding complexes consisting of NF-κB family members, as labeled, were identified by antibody supershift analysis. (0.67 MB TIF) [file pone.0011875.s002.tif]
